# Efficacy of Danzhixiaoyao tablets combined with methylcobalamin tablets in the treatment of burning mouth syndrome: an open-label, randomized controlled trial

**DOI:** 10.1186/s12903-024-04318-2

**Published:** 2024-05-24

**Authors:** Ya Gao, Jingwen Yang, Huimin Sun, Haiwen Zhou

**Affiliations:** 1 Department of Oral Mucosal Diseases, Ninth People’s Hospital，Shanghai Jiao Tong University School of Medicine, Shanghai, China; 2 School of Stomatology, Shanghai Jiao Tong University, Shanghai, China; 3 National Center for Stomatalogy, Shanghai, China; 4 National Clinical Research Center for Oral Diseases, Shanghai, China; 5 Shanghai Key Laboratory of Stomatology, Shanghai, China; 6 Shanghai Research Institute of Stomatology, Shanghai, China

**Keywords:** burning mouth syndrome, Danzhixiaoyao pills, Chinese patent medicine, Chinese traditional treatment, VAS, Traditional Chinese medicine(TCM), Syndrome, Depression, Anxiety

## Abstract

**Objectives:**

This randomized controlled trial compared the efficacy and tolerability of danzhixiaoyao pills in the accurate treatment of patients with burning mouth syndrome (BMS).

**Method:**

Collect a total of 78 patients (75 female patients and 3 male patients) from the oral mucosa department who were considered eligible fromOctober 2020 to October 2022.The patients were randomized and divided into trial group and control group.The trail group received danzhixiaoyao pills and mecobalamine tablets while the control group was given mecobalamine tablets.The Visual Analogue Scale (VAS), Beck Anxiety Inventory(BAI), Beck Depression Inventory (BDI), Oral Health Impact Profile (OHIP-14), Traditional Chinese medicine(TCM) syndrome integral and adverse reactions were performed at baseline and after 2, 4, and 6 weeks of treatment. Descriptive statistics, including the Wilcoxon rank-sum test and the Chi-square test for median comparisons between different times, were used.

**Result:**

1.After treatment, the VAS, BDI,OHIP-14, and TCM syndrome integral in the trial group had a significant decrease than the control group(*P*< 0.05).However, there was no statistical difference in the BAI scores between the two groups (*P*> 0.05). 2.According to the efficacy determination criteria , the total effective rate of the test group was 73.68% , the control group was 52.94% and the recurrence rate was 0. There was a significant difference between the two groups (Z=-2.688, *P* < 0.05). The results showed that the curative effect of test group was better than that of control group.3. No adverse effects occurred in patients in either group.

**Conclusion:**

Danzhixiaoyao pills has demonstrated to have a positive effect in relieving BMS symptoms and in improving a patient's overall quality of life with no AEs compared with the control group. The efficacy evaluation systems that can be verified and complementary in this study provide a perfect, effective and referential evaluation system for the use of Chinese patent medicine in the treatment of oral mucosal diseases.

**Trial Registration:**

Registry name: Chinese Clinical trail Registry

Registration number: ChiCTR2000038189

Date of Registration: 2020-09-13

Please visit (https://www.chictr.org.cn/showproj.html?proj=61462) to the protocol.

## Introduction

Burning Mouth Syndrome (BMS) is a syndrome characterized by Burning pain in the tongue. It can be accompanied with different symptoms such as dry mouth, abnormal sensation and taste change, and often without obvious clinical signs of damage, no characteristic histopathological changes. Women before and after menopause are more common with BMS, often accompanied by obvious psychological factors [1] . According to epidemiological research on BMS, its prevalence ranges from 0.7% to 15% [2]. The average age of onset is between 55 to 60 years old, with rare cases occurring in individuals below 30 years old. The male-to-female ratio of affected individuals is between 1:3 to 1:17. Moreover, over 90% of female patients experience BMS during perimenopause and postmenopause [3–5]. Due to the complex etiology and unclear pathogenesis of burning mouth syndrome (BMS), there are currently no standard treatment methods available. Treating BMS is challenging, and it remains a difficult-to-treat condition. Modern medicine suggests that the etiology and pathogenesis of burning mouth syndrome (BMS) may be associated with local factors, systemic factors, psychological factors, and neurological disorders. Current treatment methods include oral antihistamines, antidepressants, vitamins, hormone replacement therapy, local nerve block anesthesia, electrical stimulation, low-level laser therapy, and cognitive-behavioral therapy [6–12], but the therapeutic effects are not satisfactory [13–16]. Therefore, it is urgent to actively seek effective drugs for treating burning mouth syndrome, which has important clinical significance.

According to the analysis based on traditional Chinese medicine theory, the pathogenesis of this condition is often related to liver and kidney yin deficiency, spleen dysfunction in transforming and transporting, deficiency fire flaring up, qi stagnation and blood stasis, blood stasis obstructing the meridians, and qi deficiency unable to promote the circulation of menstrual blood, leading to qi deficiency and blood stasis. Tongue pain and numbness are both symptoms of blood stasis, which causes pain when the flow is blocked. Therefore, the treatment for this condition should focus on soothing the liver, strengthening the spleen, resolving stagnation, and calming the mind.

Xiaoyao San, derived from "Formulary of Peaceful Benevolent Dispensary," is a representative prescription used for soothing liver, relieving depression, nourishing blood, and strengthening spleen. Based on this, the combination of Cortex Moutan and gardenia is used to create Danzhi Xiaoyao San, helping in nourishing the blood, strengthening the spleen, soothing the liver, and clearing heat [2, 3]. Xiaoyao San, also known as Rambling Powder, is widely used in clinical practice. It is affordable and safe in terms of pricing [17–22].

This study adopts a randomized, controlled research method to investigate the clinical efficacy of oral Danzhi Xiaoyao tablets combined with methylcobalamin tablets in the treatment of burning mouth syndrome compared to the effects of oral methylcobalamin tablets alone.This study aims to explore the efficacy and safety of Danzhixiaoyao tablet in treating BMS through randomized controlled trials. The goal is to develop effective treatment strategies with high success rates, leading to advanced diagnostic and therapeutic guidelines for both domestic and international use.

## Material and methods

### Study design, participants, and data collection

#### Patients

This was an open-label, prospective, randomized, interventional study, carried out at the outpatient department of oral mucosa, the ninth people's Hospital affiliated to “Shanghai Jiao Tong University School of Medicine”, recruting patients suffering from BMS. The study began in October 2020 and finished in October 2022. It was conducted in accordance with the ethical principles of the World Medical Association Declaration of Helsinki and was approved by the Ethical Committee of the Hospital (Approval Number: SH9H-2020-T169-1).It was registered in the ChiCTR with the registration number: ChiCTR2000038189. The full date of first registration is 13/09/2020. All the patients provided their written informed consent for the management of personal data before participating. The nature of the drugs used for the treatment, the mechanisms of action, and the possible AEs were explained to the patients involved. 300 yuan payment was provided for participation in the study.

Patients' study eligibility was assessed at baseline on the basis of the following inclusion and exclusion criteria; the inclusion group included patients with BMS who were receiving their first consultation and had never been treated for ADs [9].

The BMS group inclusion criteria were the following:

1. Over 18, under 75.
2. Gender is not limited.
3. Meets the International Classification of Orofacial Pain, 1st edition (IHS, 2018):
  a patients experiencing continuous symptoms of oral burning or pain persisting for at least 2 hr per day, lasting for longer than three months, with no paroxysms and not following any unilateral nerve trajectory;
  b Patients not presenting any clinical mucosal alterations;
  c Patients reporting normal blood test findings (including blood count, blood glucose levels and glycated hemoglobin, serum iron, ferritin and transferrin, folic acid, and vitamin B12 levels);
  d Patients having a body mass index (BMI) of less than 30;
  e Patients not having undergone any previous treatment with systemic psychotropic drugs.
4. Conformed to syndrome following [2]:
  a Irritability, or sweating night sweats, or headache eye astringency, or cheeks red mouth dry, or irregular menstruation, less abdominal distension pain, or urination astringent pain;
  b The tongue is red and the fur is yellow
  c Pulse strings or imaginary numbers.
  d Had not received any treatment for BMS in 2 weeks.
5. The visual analogue scale (Vas) was greater than or equal to 2.

The BMS group exclusion criteria were as follows:

1. Patients having other oral mucosal diseases.
2. Patients having systemic diseases, such as diabetes, anemia, nutritional deficiency (vitamins, folic acid, Fe, Zn, etc.), abnormal thyroid function, allergy to food or dental materials, autoimmune diseases, psychiatric diseases, Sjogren's syndrome, etc.
3. Patients having bad oral habits, such as cheek biting and tongue extension.
4. Patients having local stimulating factors, such as sharp tooth tips, poor restorations, and chemical and physical injuries.
5. Patients do not meet the syndrome of deficiency of fire clip caused by stagnation of liver qi.
6. Pregnant or breastfeeding women.

#### Observational indicators

1. Main indicators:

Visual Analogue Scale (VAS) [4, 5, 13]: The VAS pain score is an internationally recognized and universal method of visual simulation score. Operation method: Patients will self-evaluate their clinical symptoms on a 10-cm visual ruler. 0 Represents normal, no pain, and 10 is extremely uncomfortable and very painful. Higher scores responded to a higher pain grade.

#### Secondary indicators

1. Beck Depression Inventory (BDI) [14, 15]. The BDI is a more commonly used inventory in the clinical evaluation of depression status. The entire scale consists of 21 sets of items with 4 statements for each set, and a graded number of previous Arabic numerals is marked for each sentence. After all 21 groups are finished, the delineated scores of each group are added up to get the total score. Based on the total score, know if there is depression, how much the degree of depression.

Beck Anxiety Inventory (BAI)[16]. The BAI, compiled by Alonbeck and other scholars in 1985, is a self-rating scale with 21 items. The scale was scored with level 4 to assess how well subjects are disturbed by multiple anxiety symptoms. For adults with anxiety symptoms. It can accurately reflect the subjective anxiety.

Oral Health Impact Profile-14 (OHIP-14) [23]. This table is a widely used scale in the field of oral related quality of life research, reflecting the impact of oral diseases and prevention and treatment on patients' physiological function, mental spirit and social activities.

Traditional Chinese Medicine Syndrome Integral Score (TCMSIS). This study refers to the “2002 Guiding Principles for Clinical Research of New TCM Drugs”, and according to the efficacy evaluation criteria of patients' clinical signs and BMS efficacy research literature, the comprehensive evaluation method of BMS TCM syndrome points was formulated.

Clinical symptoms of BMS are divided into main symptoms and secondary symptoms:

Main symptoms: burning mouth or tough; irritable; headache and eye astringent; flushed cheeks and dry mouth.

Secondary symptoms: irregular menses and lower abdomen pain; painful urination; coating on the tongue; pulse condition.

Scoring evaluation method: a semi-quantitative grade scoring method was used. Main symptoms were graded as none (0), mild (2), moderate (4), and severe (6). Secondary symptoms were graded as none (0), mild (1), moderate (2), and severe (3).The integral score of TCM syndrome is shown in Table 1.

**Table 1:** Traditional Chinese Medicine Syndrome Integral Score

Project	Standards	Points
Burning mouth or tough	painless (VAS score of 0-1)	0
	mild pain (VAS score of 2-4)	2
	moderate pain VAS score of 5-8	4
	severe pain VAS score of 9-10	6
Irritable	No	0
	Occasional emotional irritability	2
	Easy to be irritable	4
	Often irritable and difficult to self-control	6
Headache and eye astringent	No	0
	Occasionally, not affect the normal activities and work	2
	Repeatedly, can insist on normal work	4
	Often appear, difficult to insist on normal work	6
Flushed cheeks and dry mouth	No	0
	Occasionally	2
	Repeatedly	4
	Often appear	6
Irregular menses、lower abdomen pain	No	0
	Occasionally, not affect the normal activities and work	1
	Repeatedly, can insist on normal work	2
	Often appear, difficult to insist on normal work	3
Painful urination	No	0
	Occasionally	1
	Repeatedly	2
	Often appear	3
Coating on the tongue	Normal	0
	Red tough with yellow or thin coating,the tongue is swollen or has tooth marks	1
Pulse condition	Normal	0
	Thready and rapid pulse	1

All scales were checked for completeness before collection and administered by the same clinician to reduce inter-individual differences in judgment

## Establish the evaluation system of BMS medicine efficacy

This study developed a new BMS Western medicine efficacy evaluation system, including VAS pain score, BAI score, BDI score, BDI score and OHIP-14 score, to evaluate the efficacy of subjects in terms of oral burning pain symptoms, anxiety, depression, oral health impact degree. Clinical response to treatment was defined as:

Cure: Oral discomfort symptoms completely disappeared, BDI scores were <7 points, BAI scores were<45 and OHIP-14 scores were <14 points.

Efficacy: The pain efficacy index was 60%; BDI score decreased by one grade, BAI scores were<45 and OHIP-14 score decreased by 14 points or less than 14 points.

Effective: burning mouth and tongue pain and discomfort decreased, and the pain efficacy index of 20%: BDI, BAI, and OHIP-14 scores decreased.

Ineffectiveness: burning mouth and tongue pain is not reduced, the pain efficacy index is <20%; BDI, BAI and OHIP-14 scores were not decreased or increased.

Pain efficacy index = (pre-treatment pain score-post-treatment pain score)/pre-treatment pain score × 100%

Total effective rate = (number of cured persons + number of obvious effect persons + number of effective persons)/total number of persons × 100%

### Treatment protocol

All the patients(78) were randomized into trial and control groups, with 39 cases each.

1. The trial group that included patients treated with Danzhi Xiaoyao tablets (2.8g each time, 2 times a day) and methylcobalamin tablets (0.5mg each time, 3 times a day).
2. The control group that included patients treated with methylcobalamin tablets(0.5mg each time, 3 times a day).

Danzhi Xiaoyao tablet is produced by Hunan Tianji Caotang Pharmaceutical Co., Ltd., with dosage form: 0.35g * 12 tablets * 5 board. Methylcobalamin tablets made by Eisai Pharmaceutical Co., Ltd., the dosage form: 0.5mg * 10 pieces * 2 boards / box.

The patients were observed for 6 weeks including 4 weeks of medication and 2 weeks of observation after enrollment, attending a total of five appointments: baseline (time 1) and after 1day (time 2), 2 weeks (time 3), 4 weeks (time 4), and 6 weeks (time 5) postbaseline. At admission, the following information was recorded: age, gender, job status, social habits, disease duration (in years), social habits, oral symptoms, systemic diseases, and drug consumption.

**Fig. 1:**
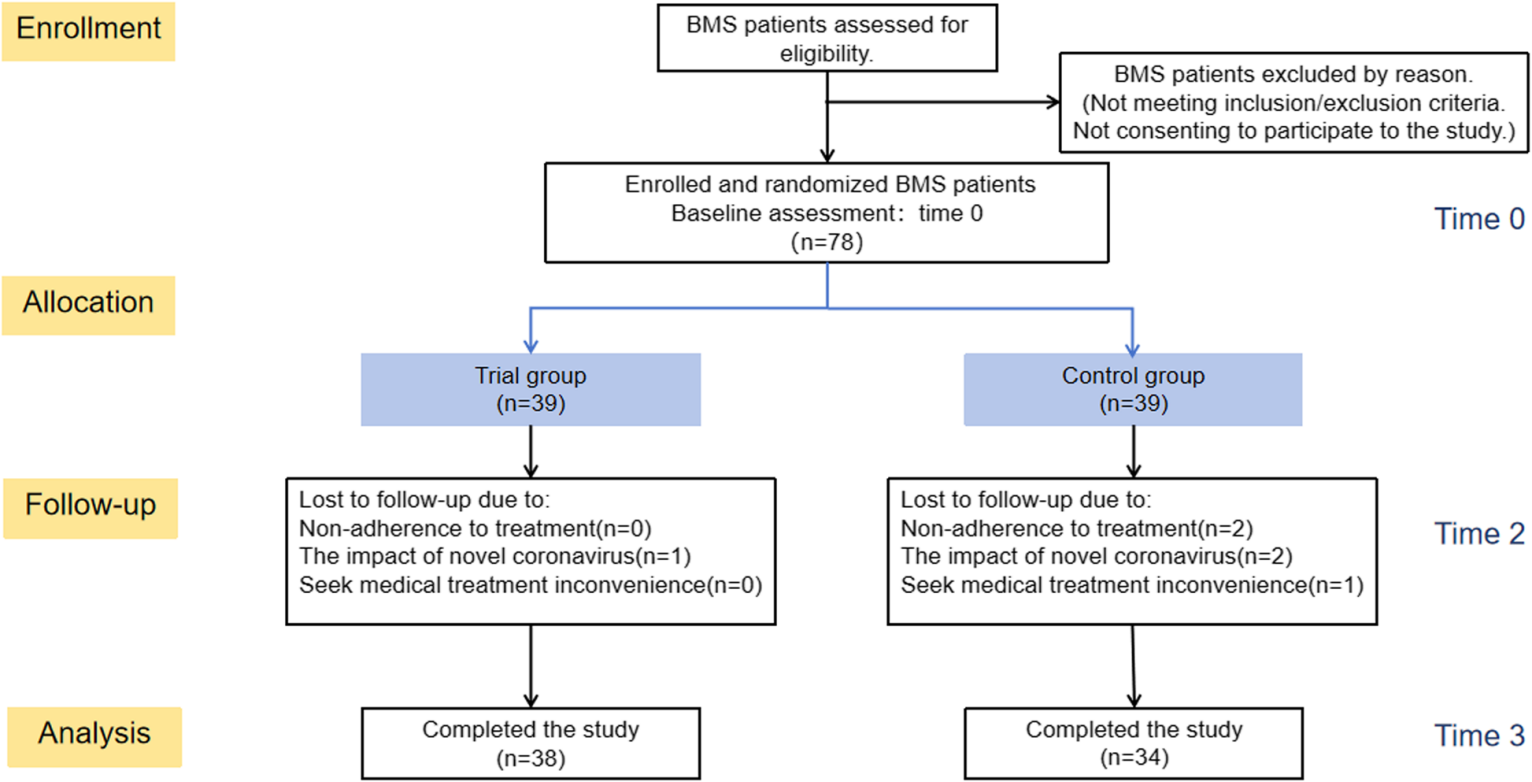
Flow chart of the study

VAS, BDI, BAI, OHIP-14 and TCMSIS were administered at baseline (Time 0) and at time 1,2,3,(after 2,4,6 weeks, respectively) . At baseline and at time 2, regular safety measures including BMI, blood pressure, blood tests, and electrocardiogram (ECG) assessments were performed. If there are any clinically significant changes in these safety indicators during treatment, the study should be stopped and appropriate treatment should be carried out[8, 9].The specific visit time points and the recorded time points of this study are shown in Fig. 1.

### Data analysis

The sample size was calculated using PASS15 software. Based on clinical preliminary experiments and literature data, the estimated effective rate in the experimental group is 70%, while in the control group it is 35%. Assuming that the effective rate in the experimental group is greater than that in the control group, with a one-sided type I error rate of 0.05 and a power of 0.80, each group requires 32 participants. Assuming a dropout rate of 20%, each group should have at least 39 participants, resulting in a total of 78 participants.

Randomization method: Random number table method. The random numbers are generated using the "=Rand()" function in Microsoft Excel software and assigned consecutive numbers (1-78) to correspond to the subject numbers. The random numbers are then sorted in ascending order, with the first 39 numbers assigned to the experimental group and the remaining 39 numbers assigned to the control group. The statistical analysis was conducted using SPSS version 22. Significance of results and differences was determined as moderately or strongly significant when p-values were below 0.05 or 0.01, respectively. Descriptive statistics, such as means and standard deviation, were employed to analyze age, gender and clinical characteristics across treatment groups. Frequencies and percentages for all categorical variables were calculated. The differences between the two groups were assessed using the independent samples t-test. The effect of treatment on the dependent variables over time was analyzed by comparing the means of VAS, BDI, BAI, OHIP-14, and TCMSIS scores at times 1,2, and 3 with the Chi-square test.

## Results

A total of 78 cases were collected. There were 39 cases in the trial group and 39 cases in the control group, 38 cases in the final trial group were completed and 1 case was lost to follow-up, and 34 cases in the control group were completed and 5 cases were lost to follow-up.

The datasets analysed during the current study are not publicly available due to sensitive patient information and personal data, which cannot be shared due to privacy and ethical considerations but are available from the corresponding author on reasonable request.

### Baseline

There was no significant difference in gender composition, age, VAS, BDI, BAI, OHIP-14 and TCM syndrome score before treatment between the two groups (p < 0.05) , which are summarized in Tables 2,3 and 4.

**Table 2:** Comparison of gender composition between the two patient groups

Group	Number(n)	Male(n)	Female(n)	*P*
Test group	39	2	37	0.56
Control group	39	1	38	

The significant difference was measured by the Chi-square test

**Table 3:** Comparison of patient age composition between the two groups

Group	Number(n)	Age interval(y)	Age(‾x±s)	*P*
Test group	39	29-78	55.39±9.63	0.74
Control group	39	31-79	57.01±10.27	

The significant difference among means was measured by the independent samples t-test

**Table 4:** Comparison of the pre-treatment evaluation indicators in the two patient groups

Evaluating indicator	Group	Number(n)	Score(x±s)	*P*
VAS	Test group	39	5.34±1.54	0.12
	Control group	39	5.71±1.74	
BDI	Test group	39	12.76±8.69	0.43
	Control group	39	14.38±10.21	
BAI	Test group	39	31.23±7.96	0.06
	Control group	39	31.72±7.99	
OHIP-14	Test group	39	14.37±10.15	0.20
	Control group	39	15.72±9.38	
TCM syndrome	Test group	39	13.61±3.61	0.08
	Control group	39	14.28±4.067	

The significant difference among means was measured by the independent samples t-test

*Abbreviations*: *VAS* Visual Analogue Scale, *BDI* Beck Depression Inventory, *BAI* Beck Anxiety Inventory, *OHIP-14* Oral Health Impact Profile-14, *TCM* Traditional Chinese Medicine

### Statistical analysis of the clinical efficacy

Statistical comparison results at different time points in the two groups are summarized in Table 5.

**Table 5:** Statistical comparison results at different time points in the two groups

Evaluating indicator	Group	2 Weeks(*P*)	4 Weeks(*P*)	6 Weeks(*P*)
VAS	Test group	0.001**	0.000**	0.000**
	Control group	0.021*	0.002**	0.000**
BDI	Test group	0.017*	0.013*	0.005**
	Control group	0.020*	0.046*	0.005**
BAI	Test group	0.379	0.002**	0.000**
	Control group	0.131	0.037*	0.003**
OHIP-14	Test group	0.444	0.004**	0.011*
	Control group	0.746	0.473	0.078
TCM syndrome	Test group	0.000**	0.000**	0.000**
	Control group	0.008**	0.001**	0.000**

The significant difference among means was measured by the Chi-square test

*Abbreviations*: *VAS* Visual Analogue Scale, *BDI* Beck Depression Inventory, *BAI* Beck Anxiety Inventory, *OHIP-14* Oral Health Impact Profile-14, TCM:Traditional Chinese Medicine

^*^Significant .01 < *p* ≤ .05

^**^Significant *p* ≤ .01

#### Comparison of the VAS scores before and after treatment between the two patient groups

The VAS score of each time-node trail group was lower than that of the control group, and the results were statistically different (*P* <0.05), indicating that the efficacy of the trail group was better than that of the control group (Table 6).

**Table 6:** Comparison of VAS scores before and after treatment between both groups

Time	Group	Score(‾x±s)	*P*
Time 0	Test group	5.40±1.56	0.262
	Control group	5.84±1.74	
Time 1	Test group	4.03±1.87	0.041*
	Control group	4.88±1.59	
Time 2	Test group	3.18±2.31	0.017*
	Control group	4.41±1.89	
Time 3	Test group	2.91±2.24	0.012*
	Control group	4.16±1.82	

The significant difference among means was measured by the independent samples t-test

^*^Significant .01 < *p* ≤ .05

#### Comparison of the BDI scores before and after treatment between the two patient groups

The BDI score of each time-node trail group was lower than the control group, and the results were statistically different (*P* <0.05), indicating that the efficacy of the trail group was better than the control group (Table 7).

**Table 7:** Comparison of BDI scores before and after treatment between both groups

Time	Group	Score(‾x±s)	*P*
Time 0	Test group	11.76±8.69	0.113
	Control group	14.56±10.52	
Time 1	Test group	6.13±5.39	0.010**
	Control group	9.65±5.86	
Time 2	Test group	5.95±7.91	0.027*
	Control group	10.06±7.46	
Time 3	Test group	3.79±4.27	0.001**
	Control group	8.24±6.57	

The significant difference among means was measured by the independent samples t-test

^*^Significant .01 < *p* ≤ .05

^**^Significant *p* ≤ .01

#### Comparison of the BAI scores before and after treatment between the two patient groups

The BAI scores of the two groups decreased at each time node after medication, but the results were not statistically different (*P*> 0.05), indicating little difference in efficacy between the two groups (Table 8).

**Table 8:** Comparison of BAI scores before and after treatment between both groups

Time	Group	Score(‾x±s)	*P*
Time 0	Test group	31.24±7.96	0.511
	Control group	32.41±7.02	
Time 1	Test group	29.79±6.18	0.810
	Control group	30.12±5.22	
Time 2	Test group	25.76±7.26	0.070
	Control group	28.53±8.01	
Time 3	Test group	25.47±7.25	0.143
	Control group	27.78±5.12	

The significant difference among means was measured by the independent samples t-test

#### Comparison of the OHIP-14 scores before and after treatment between the two patient groups

The OHIP-14 scores of the trial group were lower than those of the control group at time 1 and time 3, but the difference was not statistically significant (*P* > 0.05). This suggests that the trial group's effectiveness was not significantly different from that of the control group. However, at time 2, both groups showed a statistically significant difference in OHIP-14 scores (*P* < 0.05), indicating that the trial group performed better and was more effective than the control group (Table 9).

**Table 9:** Comparison of OHIP-14 scores before and after treatment between both groups

Time	Group	Score(‾x±s)	*P*
Time 0	Test group	14.37±10.15	0.479
	Control group	16.03±9.58	
Time 1	Test group	12.68±8.9	0.354
	Control group	15.29±9.08	
Time 2	Test group	8.39±7.14	0.005**
	Control group	14.47±8.19	
Time 3	Test group	9.26±7.31	0.201
	Control group	12.03±7.42	

The significant difference among means was measured by the independent samples t-test

^**^Significant *p* ≤ .01

#### Comparison of TCM syndrome points before and after treatment between the two patient groups

The TCM syndrome score of each time-node trail group was lower than the control group, and the results were statistically different (*P* <0.05), indicating that the efficacy of the trail group was better than the control group (Table 10).

**Table 10:** Comparison of TCM syndrome before and after treatment between both groups

Time	Group	Score(‾x±s)	*P*
Time 0	Test group	13.61±3.61	0.431
	Control group	14.32±4.08	
Time 1	Test group	10.16±3.11	0.046*
	Control group	11.77±3.59	
Time 2	Test group	9.08±3.73	0.05*
	Control group	10.94±4.19	
Time 3	Test group	7.00±3.55	0.003**
	Control group	9.74±4.07	

The significant difference among means was measured by the independent samples t-test

^*^Significant .01 < *p* ≤ .05

^**^Significant *p* ≤ .01

#### Comparison of medicine effect between two groups (total effective rate)

The total effective rate was 73.68% in the trial group and 52.94% in the control group. By rank sum test, there was statistical significance between the two groups (Z = -3.540, *p* < 0.01) , indicating that the therapeutic effect of the trial group was better than that of the control group (Table 10).

### Safety results

Any AEs were not recorded at each group.

## Discussion

Burning mouth syndrome is one of the most common oral mucosal diseases, and the number of patients with burning mouth syndrome has been increasing over the past 20 years. According to statistics, our department has seen an average of 10,000 visits of burning mouth syndrome patients per year from 2018 to 2020, accounting for 10% of the department’s annual visits. Burning mouth syndrome ranks third in terms of the number of visits for specific diseases. Due to the lack of standardized treatment guidelines, patients have to endure the torment of pain for a long time, often experiencing unbearable suffering. Severe cases may even feel a desire to end their lives, severely impacting patients’ emotional well-being and sleep, and affecting their overall quality of life. Therefore, actively searching for effective medications to treat burning mouth syndrome is an urgent priority and holds significant clinical significance (Table 11).

**Table 11:** Clinical effect in the two groups (total response efficiency)

Group	Recure(n, %)	Excellent(n, %)	Effective(n, %)	Noneffective(n, %)	Total effective rate (n, %)
Test group	10 (26.31)	5 (13.16)	13 (34.12)	10 (26.31)	28 (73.68)
Control group	1 (2.94)	3 (8.82)	14 (41.18)	16 (47.06)	18 (52.94)

Through extensive literature review, we found that Xiaoyaosan is commonly used in clinical practice [17–22]. However, there are only 5 reports on its application in BMS. These reports mainly focused on modifying the decoction of Xiaoyaosan, including 1 case using Xiaoyao powder [24] and 4 cases using danzhi xiaoyaosan [25–27]. Although there were significant differences in clinical outcomes between the two groups in these studies, the sample selection, trial design, and evaluation of efficacy were not scientifically conducted. Hence, it is important and valuable to utilize scientific clinical experimental research methods to further investigate the clinical efficacy of Xiaoyaosan in treating burning mouth syndrome.

Danzhixiaoyaosan, with the prescription by Chinese thorowax, angelica, white peony root, tylo, poria cocos, licorice, mint, burning ginger, licorice, cortex Moutan, fructus gardeniae, has the role of nourishing blood and spleen, dredging liver and clearing heat [28].

Currently, there is a Chinese patent medicine called Danzhi Xiaoyao tablets available on the market. It is formulated based on the classic traditional Chinese medicine prescription Danzhi Xiaoyao San and has the effects of soothing the liver, invigorating the spleen, and regulating blood circulation. Compared to traditional herbal decoctions, the clinical use of Danzhi Xiaoyao tablets is more convenient and cost-effective.In recent years, the study group has used modified Xiaoyao tablet to treat more than 2000 cases of BMS. The pre-experiment has been carried out, and the clinical effect is remarkable. The burning pain of the patients is obviously relieved or even disappeared after taking the medicine.

In this project, an open-lable, randomized, controlled observational study have been

conducted to investigate the clinical efficacy of oral Danzhi Xiaoyao tablets combined with methylcobalamin compared to oral methylcobalamin alone in the treatment of burning mouth syndrome. The study aims to standardize the use of individualized herbal medicine prescriptions and explore an effective medication for treating burning mouth syndrome, providing scientific and reliable experimental evidence for future clinical promotion. By leveraging the strengths and advantages of traditional Chinese medicine, it is of great social significance and economic value to relieve the suffering of patients.

## Efficacy evaluation and analysis

The efficacy evaluation and analysis included the main indicators VAS score, the secondary indicators BDI, BAI , OHIP-14 and TCM syndrome score.

The main outcome measure of this study was VAS score. The results showed that the VAS score of the trial group was lower than that of the control group at each visit (*p* < 0.05). The VAS pain grades of both groups were“Moderate pain”. After 4 weeks of treatment(time 2), the trail group was reduced to“Mild pain”, while the control group was still“Moderate pain”, the results showed that the medication could effectively reduce the pain grade of the experimental group. Therefore, Danzhi Xiaoyao tablet can relieve the pain in the mouth of BMS patients. The relapse rate of the two groups was 0 at time 3 indicating that the drug was still effective and the pain did not recur after 2 weeks of drug withdrawal.

In the pathogenesis of burning mouth syndrome (BMS), psychological disorders are often mentioned. It has been proven to be associated with elevated levels of depression, anxiety, hypochondriasis, cancer phobia, and emotional instability [29, 30]. The results showed that the BDI score of the trial group was lower than that of the control group at each time point after treatment (*p* < 0.05) , but the BAI score had no significant difference (*p* > 0.05) . These results suggest that Danzhi Xiaoyao tablet can effectively improve the depression of patients with BMS, but it has no obvious effect in improving anxiety.

What is the reason for the poor performance in anti-anxiety? In clinical, we observed some patients with anxiety in the trial drug Danzhixiaoyao tablet 2,3 months after the emergence of anxiety symptoms relief. At the 4th week of treatment, the decrease of BAI in the experimental group was greater than that in the control group (5.48 > 3.88). It is suggested that Danzhi Xiaoyao tablet can improve the anxiety of patients with BMS. The lack of significant differences between the two groups may be due to the short duration of the study design and the need to extend the observation period in terms of improving anxiety.The living environment of the subjects is complex, and various social factors may affect the prognosis of the disease. The pain and discomfort caused by the disease and the quality of life decline will also affect the patient's emotional changes. Various social life stress events and long-term social life stress are important in the development of chronic pain [24]. During the observation period, we found that there were 3 patients in the trail group and 2 patients in the control group, when they had anxiety and depression during the course of taking medicine, the symptoms of oral pain would be aggravated. After treatment, the scores of anxiety and depression were significantly decreased, and the symptoms of oral pain were also significantly relieved. Due to the differences in personality characteristics of the subjects, the self-regulation ability of negative emotions is uneven, which may lead to the subjects can not successfully self-adjust their emotions in the whole course of the short study, this is also one of the important reasons why Danzhi Xiaoyao tablet can not improve the anxiety symptoms obviously.

Health-related quality of life is an important goal and outcome of patients after illness, and as part of overall health, oral health impact scale(OHIP) has been widely used and recognized in the past decades. OHIP reflects the potential impact of oral diseases (including dental, periodontal, mucosa, tumor, etc.) on quality of life, including functional limitations, physical pain, mental discomfort, physical disability, mental disability, social disability, and disability 7 areas. It has been proved to be reliable and effective, and is more suitable for clinical application. In this study, there was no significant difference in OHIP-14 scores between the two groups after 2 weeks(time 1) of treatment and 2 weeks after withdrawal(time 3) (*P* > 0.05) , but after 4 weeks of treatment(time 2), the OHIP-14 scores of the two groups were significantly higher than that of the control group (*p* < 0.05) , there was statistical significance (*p* < 0.05) . Therefore, Danzhi Xiaoyao tablets can improve the oral health-related quality of life in patients with significant effect. At time 3, some subjects score increased, indicating that the medication time is not enough Therefor we should increase the medication time, continue to use the drug.

The results of this study showed that the mean value of the TCM syndrome score in the post-medication trail group at each time node was lower than that of the control group (*P* <0.05), showing the better efficacy of the trail group than the control group. According to the efficacy determination criteria of TCM syndrome, the total response rate of the trail group was 63.16%, the control group was 47.06%, and the trail group was better than the control group (Z= -5.157, *P* <0.05). It can be seen that Danzhi Xiaoyao tablets can effectively improve the TCM syndrome in patients with BMS deficiency.

## Establishment of a BMS efficacy evaluation system

The evaluation of therapeutic efficacy is a crucial aspect of clinical research. However, there is currently no standardized criterion to assess the effectiveness of treatment for Burning Mouth Syndrome (BMS). Upon reviewing the literature on BMS clinical studies, it becomes evident that the existing evaluation systems are imperfect. Some researchers rely solely on patients' subjective assessments, categorizing outcomes as significant, effective, or ineffective based on symptomatology, or as cured, improved, or ineffective [28, 31, 32]. These evaluation criteria are vague, subjective, and lack scientific rigor, making it challenging to accurately gauge true efficacy. Given our objective of treating BMS with Chinese patent medicine, it is imperative to establish a comprehensive and objective evaluation system to assess its therapeutic effectiveness. Hence, this study proposes a novel curative effect evaluation system, aiming to provide a more robust and effective means of evaluating the treatment outcomes of oral mucosal diseases with Chinese patent medicine.

The content of the medicine evaluation system in this study includes four parts: oral symptoms, depression, anxiety, and the degree of oral health impact, which were scored through the scale. In clinical research, the International General VAS pain score is generally used as a single index to evaluate the efficacy of BMS, and only a few studies have evaluated the symptoms of anxiety and depression. However, the evaluation criteria were not included in the comprehensive efficacy analysis, but were analyzed separately in the final discussion.The clinical symptoms of BMS are not confined to the oral cavity. Patients often accompanied by anxiety, depression and other adverse emotions. The suffering caused by disease often seriously affects the quality of life of patients with BMS, so it is necessary to improve the clinical symptoms and pay attention to the mental health and quality of life of patients before and after treatment. In this study, BAI and BDI were introduced to evaluate the anxiety and depression of the subjects, and OHIP-14 was introduced to observe the changes of oral health quality of life. This study innovatively combined VAS Pain Index, BAI score, BDI score and OHIP-14 score to establish the evaluation system of Western medicine curative effect, and make a comprehensive analysis of the curative effect of patients as far as possible, instead of just evaluating pain, so we can get more realistic data.

## Limitations

This study has certain limitations. Firstly, it was an open-label trial conducted in a tertiary hospital, meaning that both the clinical doctors and patients were aware of the type of treatment being administered. Secondly, the sample size was calculated to detect differences in clinical outcomes. Although we have already treated around 2,000 patients with Danzhi Xiaoyao tablets in several years of clinical pre-experiments, the small sample size of this study may was small and could be limited to detect differences in each outcome. Thirdly, although a relatively comprehensive efficacy evaluation system has been established, the efficacy system of this study also needs to be further improved, in the course of the study we found that the VAS score can reflect the degree of pain improvement, it can not reflect the improvement of pain frequency and pain duration in some patients. There were 3 subjects in the trail group and 5 subjects in the control group who experienced accompanying symptoms of oral numbness, astringent taste, and foreign body sensation in addition to burning pain. However, the improvement of these symptoms after medication was not evaluated in this efficacy evaluation system. For these cases, the efficacy evaluation system of this study cannot be objectively recorded and evaluated, so it needs to be further improved and supplemented.

## Reasons for loss to follow-up

The successful completion of this study requires high compliance of the subjects. One patient in the trial group was lost to follow-up, and five patient in the control group was lost to follow-up, all of whom were female. The reasons are as follows:

1. poor compliance, not on time medication.
2. the impact of novel coronavirus outbreak in 2019-2022.
3. others, such as non-local patients, seek medical treatment inconvenience.

## Conclusions

The treatment of burning mouth syndrome (BMS) requires a comprehensive evaluation and should focus on individual patients and holistic management. Clinicians should work collaboratively with patients to treat oral symptoms and any associated psychological comorbidities. Despite limitations in this study, the findings suggest that Danzhi Xiaoyao tablet have good efficacy and safety in the treatment of BMS, presenting a new perspective in managing this condition and improving patients’ quality of life. For patients who show slow response, clinicians may consider adjusting the treatment course accordingly. If there is no response to Danzhi Xiaoyao tablet, alternative therapeutic medications can be considered.This study established a comprehensive evaluation system of oral burning pain, anxiety, depression and quality of life, which provided a perfect, effective and referential evaluation system for the treatment of oral mucosal diseases with Chinese patent medicines. However, in future large-scale studies, it is necessary to improve the efficacy evaluation system and include assessments of improvements in pain frequency and accompanying symptoms such as numbness. The results of this study are optimistic, but in the future, it is necessary to extend the duration of drug observation appropriately, increase the number of subjects, and conduct multicenter, double-blind, placebo-controlled trials to further refine the study results.

## Data Availability

The datasets generated during the current study are not publicly available due [the data also forms part of an ongoing study] but are available from the corresponding author on reasonable request.
